# Antimicrobial Activity of Chitosan/Gelatin/Poly(vinyl alcohol) Ternary Blend Film Incorporated with *Duchesnea indica* Extract in Strawberry Applications

**DOI:** 10.3390/foods11243963

**Published:** 2022-12-07

**Authors:** Hye-Jo Choi, Sung-Wook Choi, Nari Lee, Hyun-Joo Chang

**Affiliations:** 1 Research Group of Safety and Distribution, Korea Food Research Institute, Wanju 55365, Republic of Korea; 2 Department of Food Science and Technology, Jeonbuk National University, Jeonju 54896, Republic of Korea

**Keywords:** biodegradable coating, antimicrobial film, chitosan, gelatin, poly(vinyl alcohol), *Duchesnea indica*, strawberry

## Abstract

Chitosan (CTS)/gelatin (GEL)/poly(vinyl alcohol) (PVA)-based composite films with different concentrations of *Duchesnea indica* extract (DIE) (6.25 and 25 mg/mL), an antimicrobial agent, were manufactured using a casting technique. Results indicated that elongation at break decreased as DIE was added at higher concentrations. Composite films showed no significant differences in thickness, tensile strength, and water vapor permeability. Scanning electron microscopy images revealed that DIE was successfully incorporated into film matrices to interact with polymers. The addition of DIE to the film inhibited the growth of *S. aureus* by up to 4.9 log CFU/mL. The inhibitory effect on *S. aureus* using DIE-incorporated coating applied to strawberries was greatest at room temperature storage for 24 h only when it was coated twice or more. The maximum inhibition in strawberries was 2.5 log CFU/g when they were coated twice and 3.2 log CFU/g when they were coated three times. The results of this study suggest that DIE could be used as a natural antimicrobial agent, and DIE-integrated CTS/GEL/PVA films or coatings have potential as a food packaging alternative for preventing foodborne pathogen contamination.

## 1. Introduction

Strawberries are mainly eaten raw and have a very short shelf life after harvesting due to their high respiration rate and perishability [1]. Fresh fruits consumed with minimal processing have the potential to be carriers of pathogen transmission. In addition, pathogenic bacteria can survive long periods on fruits, posing a potential risk to consumer health [2].

As consumers become more concerned about environmental protection and food safety, edible/biodegradable coatings and films are attracting attention as alternatives to synthetic packaging [3]. The application of these biopolymers can improve product quality by selectively blocking the permeation of gases, water vapor, and solute migration, thereby preserving physical properties [4,5]. Carbohydrates, proteins, and lipids are mainly used as raw materials for biodegradable packaging and can be used alone or in combination [5,6]. Chitosan (CTS) is most often used in the manufacture of edible coatings. It exhibits intrinsic antibacterial activity, excellent film-forming ability, and oxygen-barrier properties [7]. Gelatin (GEL), a natural water-soluble protein, is mainly obtained from bovine or fish. It is rarely used alone as a food packaging material because of its brittleness [8,9]. Poly(vinyl alcohol) (PVA) is a synthetic biodegradable polymer with the advantages of being colorless and having excellent mechanical properties. However, it has the disadvantage of not being water-resistant [10].

Numerous studies have reported the use of blends to increase applications in food packaging by overcoming the limitations of using polymers alone. Chitosan combined with gelatin can improve the performance of edible films. The dense structure formed by hydrogen bonds can also improve mechanical and barrier properties of the film [11,12]. Furthermore, a composite film of natural polymer chitosan and synthetic polymer PVA can compensate for natural polymer’s low mechanical strength [13]. Many research studies have been conducted on binary composite films made of CTS, GEL, and PVA in food application [6,13,14,15]. On the other hand, the combination of the three polymers has been mainly investigated as a biologic wound healing hydrogel because it is harmless with the ability to store and release an agent [16,17]. Ghaderi et al. [18] found that a CTS/GEL/PVA blended film with an appropriate ratio can provide greater UV protection and thermal stability than CTS/PVA films, allowing application in food packaging. However, studies applying the CTS/GEL/PVA combination on food have not been reported yet.

Synthetic antibacterial agents, which have been mainly used to prolong the storage stability of food, have the possibility of side effects, so research on natural antibiotics has been attracting a lot of attention [19]. The use of plant extracts can satisfy the growing consumer demand for being safe and environmentally friendly [20]. Incorporating biopolymers with various types of natural bioactive compounds found in plants can enhance the antioxidant and antibacterial properties of the film [19,21,22,23,24]. In addition, the interaction between bioactive substances and polymeric polymers can provide controlled release during storage [25]. Various studies have been conducted on the food applications of films or coatings which incorporated plant extracts [7,19,20,26]. As far as we have investigated, the majority of studies have applied grapefruit seed extract, a representative antimicrobial agent [8,27,28,29,30,31,32]. In addition, there are many useful but underutilized native plants around us, and the ideas to utilize them are constantly needed.

*Duchesnea indica*, a rosaceae plant, is a perennial herb widely distributed in Korea and other Asian countries such as China and Japan [33]. It has traditionally been used for detoxification and anti-tumor treatment. Its principal pharmacological constituents have been proven to be tannins, ellagic acid, and phenolic compounds [33,34]. The findings of a study testing the cytotoxicity of *Duchesnea indica* methanol extract against CCD-986Sk, a skin cell, revealed that there were no evident cytotoxic or inhibitory effects on cell development at concentrations ranging from 50 to 200 μg/mL [35]. To our knowledge, no attempt has been made to incorporate *Duchesnea indica* extract into coatings or films.

In this study, we developed a CTS/GEL/PVA ternary film integrated with *Duchesnea indica* extract (DIE) and investigated the effects of different DIE concentrations on mechanical and antimicrobial properties of the film, as well as its prospective applications for active food packaging. Furthermore, the developed film was applied to strawberries with different coating times (thickness) and the antimicrobial effect on *S. aureus*, a foodborne pathogen, was investigated during storage at room temperature.

## 2. Materials and Methods

### 2.1. Materials

Whole plants of dried *Duchesnea indica* and strawberries were purchased from a local market in Jeonju, Korea. Chitosan (low molecular weight, 75–85% deacetylated), gelatin (cold water fish skin), poly(vinyl alcohol) (MW 89,000–98,000), acetic acid (glacial 100%), dimethyl sulfoxide (DMSO) and ethanol were supplied by Sigma-Aldrich (St. Louis, MO, USA). Mueller–Hinton broth (MHB), brain–heart infusion broth (BHIB), and tryptic soy broth (TSB) were obtained from Becton Dickinson (Sparks, MD, USA). Baired–Parker agar (BPA) was acquired from Microgiene (Suwon, Gyeonggi-do, Korea). Bacterial strains used in this study were *Bacillus cereus* (ATCC 14579), *Salmonella enteritidis* (ATCC 13076), *Escherichia coli* (ATCC 10536), *Staphylococcus aureus* (ATCC 25923), and *Listeria monocytogenes* (ATCC19115).

### 2.2. Preparation of Duchesnea Indica Extract

To prepare *Duchesnea indica* extract, dried samples were grounded into a powder using a blender. Thirty grams of the powder with 600 mL of 100% ethanol solution was kept for 3 days at 35 °C in a shaking incubator. After extraction, the sample was filtered through filter paper (Whatman No. 1). All extracts obtained by repeating the steps from extraction to filtration three times were concentrated using a rotary evaporator (IKA RV-10, Germany) at 40 °C. The extracts were then weighed, dissolved in DMSO, and stored at −20 °C.

### 2.3. Preparation of Films

Films were produced by means of a casting method [3,11]. Chitosan solution (3% *w*/*v*) was prepared by dissolving chitosan in 1% acetic acid with stirring overnight at room temperature. Gelatin solution (3% *w*/*v*) was prepared by dissolving gelatin in distilled water with stirring at 50 °C for 1 h. Poly(vinyl alcohol) solution (5% *w*/*v*) was prepared by dissolving PVA in distilled water with stirring at 90 °C for 2 h. Each solution of CTS, GEL, and PVA was mixed in a 1:1:1 mass ratio and stirred at room temperature for 1 h. Two different concentrations of DIE (6.25 and 25 mg/mL in DMSO) were added using the same amounts to 5% (*v*/*w*) of the CTS/GEL/PVA (CGP) solution and stirred at room temperature for 1 h. Films added with 6.25 and 25 mg/mL extracts were named CGPD1 and CGPD2, respectively. As a control, CGP was added with DMSO instead of DIE. Finally, 20 g of the mixtures were casted into petri dishes and dried in an oven at 40 °C for 24 h. The dried films were carefully peeled off and stored at 25 °C, 50% RH until further analysis.

### 2.4. Physical and Mechanical Properties of Films

#### 2.4.1. Thickness

Film thickness was measured using a digimatic indicator (Mitutoyo Corp., Japan) to the nearest 0.001 mm. Five random positions were measured for each film. Means from three film replicates were calculated.

#### 2.4.2. Mechanical Properties

Tensile strength (TS) and elongation at break (EB) were determined using a universal testing machine (Instron 5967, USA) with a 500 N load cell [36]. Films were cut into 100 mm × 20 mm strips, initial grip separation was 50 mm, and the crosshead speed was set at 50 mm/min. Measurements were performed at room temperature and averaged over five replicates for each sample.

#### 2.4.3. Water Vapor Permeability (WVP)

The water vapor permeability of film was tested using the method of Bofei et al. [37], with some modifications. The mouth of the glass bottle, containing 10 mL of distilled water, was sealed with a film using a rubber band, and then placed in an incubator at 25 °C, 50 ± 2% RH. The weight of the bottle was measured every hour for 24 h. Water vapor permeability of the film was calculated using the following equation. This experiment was replicated three times for each sample.

(1) $$\mathrm{WVTR}=\frac{\Delta w}{A\times \Delta t}$$

(2) $$\mathrm{WVP}=\mathrm{WVTR}\times \frac{d}{sp}$$

where △*w* is the weight loss (g), *A* is the transmitted film area (m^2^), △*t* is the time interval (h), *d* is the average of film thickness (mm), and *sp* is the partial water vapor pressure gradient on the inner and outer surfaces of the film (Pa).

#### 2.4.4. Scanning Electron Microscope (SEM)

The surface and cross-section of films were observed using field-emission scanning electron microscopy (JSM-7800F, JEOL Ltd., Tokyo, Japan). Samples for observing the surface were coated with platinum. For the cross-section, films were impregnated with an epoxy resin and subjected to curing and polishing processes. All samples were taken under an accelerated voltage of 5 kV.

#### 2.4.5. Fourier-Transform Infrared (FT-IR) Spectroscopy

FT-IR spectroscopy was performed to observe structural interactions of CTS/GEL/PVA ternary film incorporated with DIE. FT-IR spectra was recorded from 4000 to 400 cm^−1^ using a FT-IR spectrometer (Perkin Elmer Spotlight 400, Shelton, CT, USA). An average of sixteen scans were taken for each sample.

### 2.5. Application of Edible Coatings to Strawberries

Strawberries were selected uniformly with no damage to maturity, color, and size. The average weight of a strawberry was 13 ± 4 g. Strawberries were randomly divided into three large groups according to the number of coatings and divided into four sub-groups according to the treatment of coating solutions, including three strawberries in each sub-group.

Prior to the application of coating, strawberries were sterilized under UV by turning back and forth for 30 min each. The same edible coating method was used as in Heydari et al. [38], with a slight modification. For each treatment, strawberries were immersed in a coating solution for 1 min and dried at 25 °C for 30 min to remove excess solution. This immersion and drying process was repeated two and three times to make various layers of coating on the strawberry surface. Non-coated strawberries were used as the negative control.

### 2.6. Antimicrobial Properties

#### 2.6.1. Determination of Minimum Inhibitory Concentration (MIC) and Minimum Bactericidal Concentration (MBC) of Duchesnea Indica Extract

The minimum inhibitory concentration (MIC) value was evaluated using the macro broth dilution method for five bacteria strains [39]. *Bacillus cereus* (ATCC 14579), *Salmonella enteritidis* (ATCC 13076), *Escherichia coli* (ATCC 10536), and *Staphylococcus aureus* (ATCC 25923) were inoculated in MHB. *Listeria monocytogenes* (ATCC 19115) was inoculated in BHIB. All media were incubated overnight at 37 °C. DIE stock solution was serially diluted two-fold with DMSO and 100 µL was added into each tube containing 900 µL of MHB or BHIB. The final concentration of DIE ranged from 0.10 to 25 mg/mL. Control tubes contained DMSO instead of DIE. Finally, 5 µL of a bacterial suspension (10^6^ CFU/mL) was inoculated into each tube and incubated at 37 °C for 24 h. MIC was determined by using the lowest concentration of DIE that inhibited visible growth after incubation.

After MIC determination, 100 µL from all tubes showing no visible bacterial growth were inoculated on MHA or BHIA plate and incubated at 37 °C for 24 h. The minimum bactericidal concentration (MBC) value was determined using the concentration without any bacterial growth on the plates.

#### 2.6.2. Antimicrobial Properties of Films

Antimicrobial analysis was determined using the method of Amankwaah et al. [27] and Wang et al. [28], with a slight modification. Film samples (20 × 20 mm) were sterilized with UV light for 10 min prior to analysis. Each sample was immersed in a test tube containing 5 mL TSB supplemented with 100 µL of bacteria suspension (10^6^ CFU/mL). Cultures without films were used as controls. All tubes were incubated at 37 °C in an incubator shaker for 6 h. Samples were taken at intervals of 0, 2, 4, and 6 h. Serial dilutions of bacterial suspension were inoculated on TSA. Finally, plates were incubated at 37 °C for 24 h. All samples were run in triplicates.

#### 2.6.3. Antimicrobial Property of Strawberry Coatings

The *S. aureus* strain was grown in TSB at 37 °C overnight. One hundred microliters of the bacterial suspension adjusted to 10^6^ CFU/mL was dispensed on the surface of coated strawberries. Strawberries inoculated with bacteria were dried at room temperature for 1 h in a clean bench. All samples were stored on an incubator shelf at 25 °C, 90% RH for 3 days and antimicrobial analysis was conducted every day.

For antimicrobial analysis, 15 g of strawberry puree was placed in a Whirl–Pak bag (Nasco, USA) containing 45 mL PBS and homogenized for 5 min with a stomacher (AES chemunex easyMIX, Bruz, France). Homogenized samples were diluted serially and inoculated on BPA plates. Plates were incubated at 37 °C for 24 h. Bacterial counts were expressed as log CFU/g of triplicate experiments [40].

### 2.7. Statistical Analysis

Data were presented as mean ± SD of repeated measurements and analyzed using ANOVA with GraphPad Prism 9 software (GraphPad Inc., La Jolla, CA, USA). Tukey’s test was used to determine the differences at 5% significance level [41].

## 3. Results and Discussion

### 3.1. Mechanical Properties

The mechanical properties are an important parameter in food packaging to protect food from external damage [18,42]. Table 1 shows the results of evaluating mechanical properties of the composite film to which DIE was added at different concentrations based on CTS/GEL/PVA by measuring thickness, TS, and EB. DIE-added film was slightly thicker than the control CGP film. However, there was no significant difference (*p* > 0.05) between them. This result might be because all films were adjusted to have the same volume using DMSO regardless of the concentration of the extract, which resulted in the same polymer concentration. Similar results have been observed by Hashemi et al. [43], showing no difference in thickness between basil seed gum film (BSG) containing up to 6% *Origanum vulgare* subsp. *viride* essential oil.

Tensile strength refers to the maximum strength that a film can withstand. Elongation at break is a measure of the ability of a film to stretch [44]. TS values ranged from 17.72 to 21.59 MPa. Incorporation of DIE into the control film did not modify the TS value (Table 1). The EB was 106.43% for CGP, 87.44% for CGPD1, and 62.24% for CGPD2, which decreased sharply with increasing DIE concentration (Table 1). Such reductions in EB could be explained by the incorporation of DIE aggregates into the polymer matrix network, limiting the movement of polymer chains [19,26,45]. Films made with different blend ratios using chitosan/poly(vinyl alchohol)/fish gelatin have been reported to have higher TS (25.90–41.93 MPa) and EB (89.36–133.13%) values than those obtained in the present study [18]. However, our EB values were much higher than those of agar/alginate/collagen blend film (26.5%) [28].

### 3.2. Physical Properties

#### 3.2.1. Water Vapor Permeability (WVP)

Water vapor permeability is an important factor when choosing food packaging. A low WVP is effective in extending the shelf life of foods [29]. As shown in Table 1, WVP values ranged from 5.16 to 6.47 g·mm·m^−2^·h^−1^·Pa^−1^, showing a tendency in close agreement with thickness results of those films, although there were no significant differences between the films. The addition of DIE to control film did not affect WVP values. Similar results have been presented by Rojas-Graü et al. [41], showing that the addition of plant essential oils had no effect on the WVP of alginate–apple puree edible film. Bonilla et al. [23] also found that the presence of guarana and rosemary extracts in the gelatin film slightly increased WVP but had no significant effect.

It has been reported that the WVP of a film depends on several variables, especially thickness [46]. According to Terzioglu et al. [42], the addition of more orange peel to CTS/PVA increased thickness and WVP values, which could be explained by the water affinity of the hydrophilic film. WVP and/or thickness values obtained in the present study were much higher than those reported for other ternary composite films, for instance, agar/alginate/collagen film or chitosan/poly(vinyl alcohol)/fish gelatin film [18,28].

#### 3.2.2. Scanning Electron Microscope (SEM)

**Figure 1:**
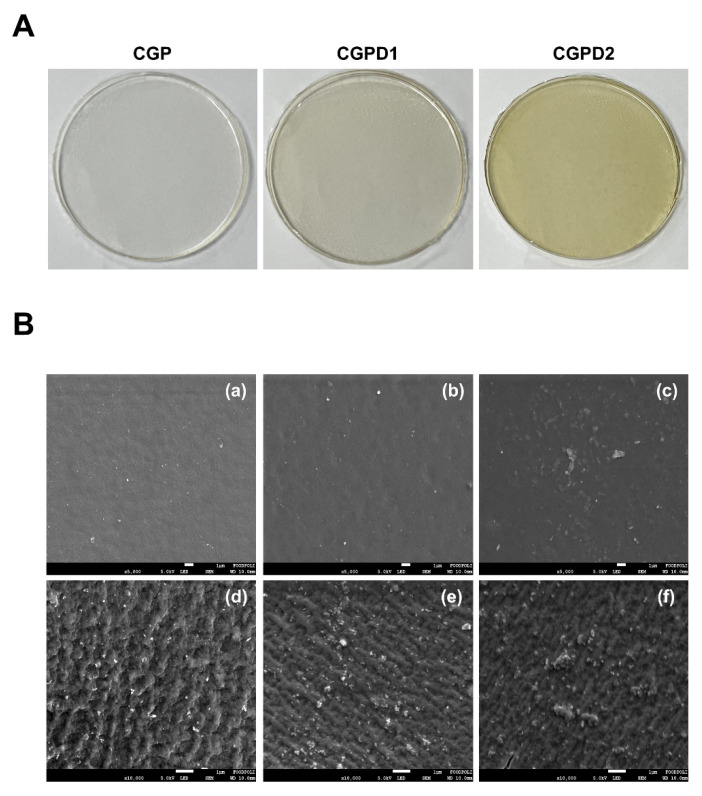
Visual appearance (**A**) and SEM images (**B**) of the surface (**a**–**c**) and cross-section (**d**–**f**) of films: (**a**,**d**) CGP; (**b**,**e**) CGPD1; (**c**,**f**) CGPD2.

The microstructure of a film depends on the interactions between its components. It also directly affects film properties [47]. DIE-incorporated CGP films were clear and transparent. They were visually homogeneous (Figure 1A). Blend films were light grey (CGPD1) or golden yellow (CGPD2) in color. Figure 1B shows SEM images of the surface and cross-section of each film. Surface morphology of all films (Figure 1B(a,b,c)) observed at 5000× magnification showed no cracks or air bubbles, indicating that DIE molecules formed a continuous network within the CGP film matrices [48]. As the extract concentration increased from 6.25 mg/mL (b) to 25 mg/mL (c), a slight grain structure appeared on the surface, which might have been due to agglomeration caused by excessive addition of the extract compound [20]. Cross-sections observed at 10,000x magnification showed a decrease in roughness as the concentration of incorporated extract increased (Figure 1B(d,e,f)). This can be explained by the redirection of polar functional groups to the top surface, which can provide an environment that allows for sustained release of an antimicrobial agent [49]. As no phase separation was observed in CGP composite films, as could be inferred from the SEM images, the three polymers were very compatible [50]. CGPD1 and CGPD2 also had different images of the two films. However, their respective morphologies were uniform, suggesting that DIE was well dispersed and integrated into CGP film matrices [30].

#### 3.2.3. Fourier-Transform Infrared (FT-IR) Spectroscopy

**Figure 2:**
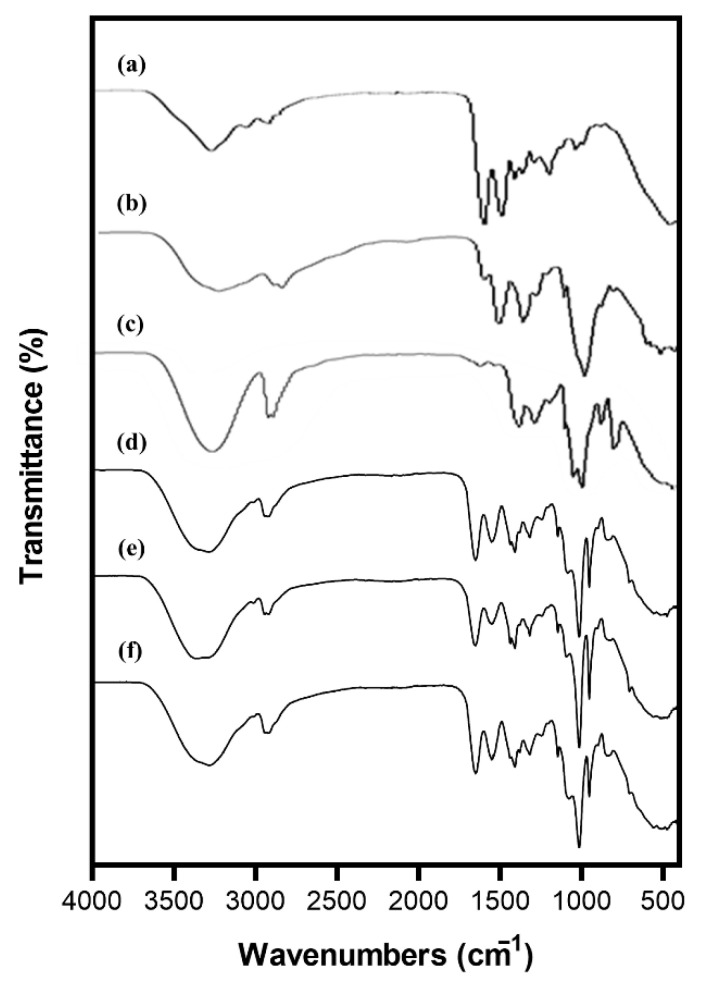
FTIR spectra of chitosan (**a**), gelatin (**b**), poly(vinyl alcohol) (**c**), CGP (**d**), CGPD1 (**e**), and CGPD2 (**f**).

FT-IR was used to evaluate the functional groups of materials and to detect possible changes in the proportion of raw materials [51]. Figure 2 shows the FT-IR spectra of each of pure polymers constituting the CGP ternary blend film and spectra of the film incorporating DIE into the CGP. For pure CTS, it presented several characteristic peaks. Absorption peaks at around 1150 and 1025 cm^−1^ corresponded to vibrations of the C-O-C bridge and C-O stretching, respectively. Amide I and amide II peaks were also found at wavelengths of about 1620 and 1525 cm^−1^, respectively. Results obtained for the chitosan film are similar to those previously reported [14,20]. The FTIR spectrum of pure GEL exhibited a peak representing an aliphatic hydroxyl group at approximately 3280 cm^−1^. Signals at about 1600 and 1500 cm^−1^ corresponded to C=O (amide I) and bending vibration of the N-H (amide II), respectively. For pure PVA, broad absorption bands near 3280 cm^−1^ were attributed to O-H stretching vibration formed by intermolecular hydroxyl groups. Two weak vibration bands between 2940 and 2880 cm^−1^ were due to the symmetric stretching of C-H. The sharp band near 1000 cm^−1^ of PVA was due to C-O stretching.

FTIR spectrum of the CTS/GEL/PVA ternary film showed characteristic absorption peaks of film components. The C=O peak at 1648 cm^−1^ shifted slightly to the left when compared to the pure GEL, which indicated hydrogen bonding interaction between PVA and GEL [52]. Furthermore, the band at 1143 cm^−1^ provided evidence for the crosslinking of PVA and the saccharide structure of CTS [24]. Fan et al. [16] reported that hydrogels composed of CTS/GEL/PVA could be inferred from peaks of 3450 and 1600 cm^−1^ representing hydrogen bonding interaction between amino and hydroxyl. All films incorporating DIE showed a pattern almost similar to the CGP spectra. The intensity of the peak changed slightly according to the concentration of DIE.

### 3.3. Antimicrobial Properties

#### 3.3.1. MIC and MBC of *Duchesnea Indica* Extract

MIC and MBC were evaluated for five microorganisms before *Duchesnea indica* extract was incorporated into the film to determine its ability as an antimicrobial film component [53]. The results (Table 2) showed that DIE had antimicrobial activities against both gram-positive (*B. cereus*, *S. aureus*, *L. monocytogenes*) and gram-negative (*S. enteritidis*, *E. coli*) bacteria. The MIC and MBC for each bacterium ranged from 0.10 to 3.13 mg/mL. *L. monocytogenes* showed the highest MIC and MBC concentration of 3.125 mg/mL, indicating the greatest resistance to growth inhibition of DIE. On the other hand, the most sensitive strain was *B. cereus*, whose value was 0.1 mg/mL for both MIC and MBC. Ilahi et al. [54] reported that MICs of *Duchesnea indica* extracted with distilled water are 12 and 10 mg/mL for *E. coli* and *S. enteritidis*, respectively. MICs in the present study were lower than those reported by Ilahi et al. [54]. These results indicate that the antimicrobial spectrum of natural plant extracts may vary depending on the part of plant used for extraction, solvents, and other experimental conditions. Considering the results obtained here, DIE is effective in inhibiting the growth of five pathogenic bacteria.

#### 3.3.2. Antimicrobial Properties of Films

**Figure 3:**
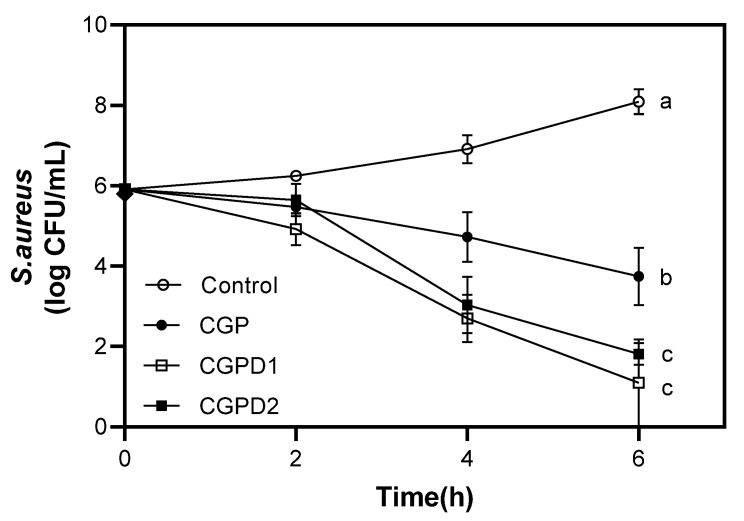
Antimicrobial property of films against *S. aureus*. The different letters indicate significant difference (*p* < 0.05). Each sample was analyzed in triplicate.

The antimicrobial activity of DIE incorporated CGP film against *S. aureus*, a major foodborne pathogen, is shown in Figure 3. All films except the control exhibited a significant inhibitory effect. However, the inhibitory mechanism is not clearly known. The control group without any film showed sustained bacterial growth since incubation, reaching from 6 to 8 log CFU/g at 6 h. CGP film decreased by 2 log CFU/g after 6 h, which might be due to the intrinsic antimicrobial activity of chitosan as previously reported [55]. One of the antimicrobial properties of chitosan against *S. aureus* is that chitosan on the cell surface can form a polymer membrane, which can inhibit nutrients from entering the cell [56]. As both CGPD1 and CGPD2 films were reduced by 4.9 and 4.2 log CFU/g, respectively, the inhibitory effect due to DIE integration was clearly noticeable. Previous studies have generally reported a positive correlation between the amount of antimicrobial substances added to the film and its antimicrobial activity [24,25,27,36]. In addition, there was no significant difference in antimicrobial activity between the two films mixed with different concentrations of DIE. These results imply that optimal, not excessive concentration of antimicrobial substances is necessary to release from film matrices and to exhibit an antimicrobial activity. Similar findings have been reported by Jang et al. [31]. They found that the antimicrobial activity of rapeseed protein–gelatin film was significantly increased with an increasing concentration of grapefruit seed extract (GSE) up to 1.0%, although the inhibition zone of the film containing more than 1.5% GSE decreased.

#### 3.3.3. Antimicrobial Property of Strawberry Coatings

**Figure 4:**
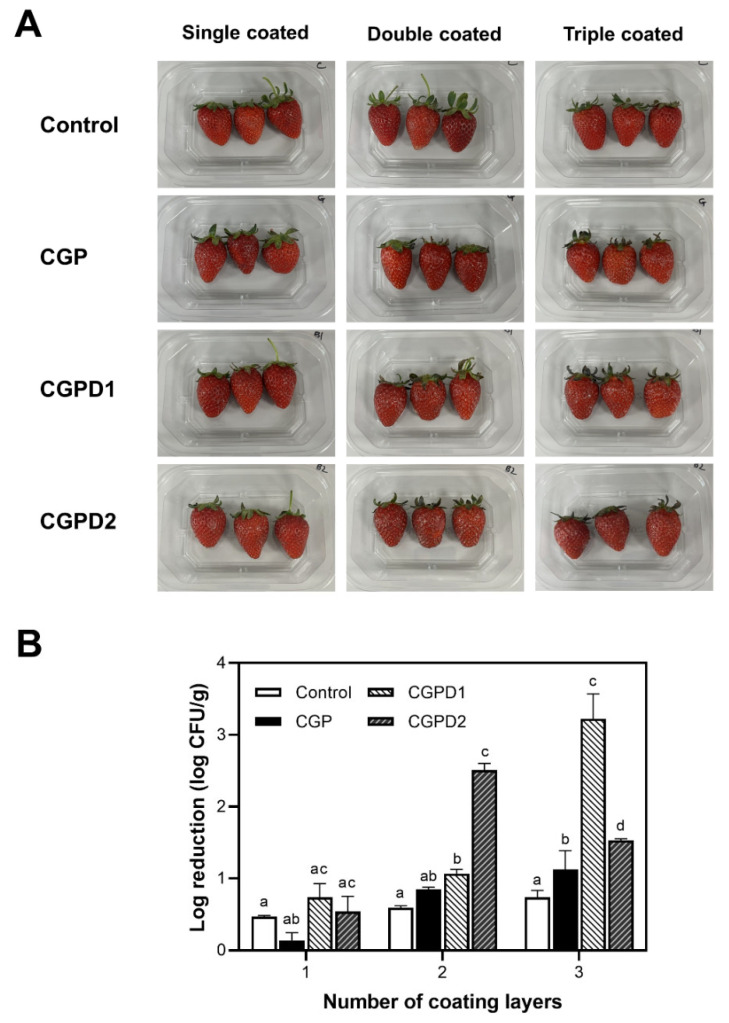
Images (**A**) and antimicrobial properties (**B**) of coated strawberry according to the number of coating layers on the first day of storage. Different small letters indicate significant differences among means in the same column (*p* < 0.05).

The appearance change of strawberries was not observed among groups of strawberry coatings after one day (Figure 4A). Figure 4B shows antimicrobial effects of strawberry coatings according to the number of coatings against *S. aureus* on day 1. During storage for 3 days, the decrease in bacterial number was the greatest on the 1st day. A similar achievement was obtained when a chitosan-grapefruit seed extract coating was applied to cherry tomatoes inoculated with *S. enteritidis*. During storage for 5 days, the highest antibacterial effect was observed immediately after coating [32]. For single-coated strawberries, all samples showed no significant difference in antimicrobial effect. This result might be because the thickness of the coating was not sufficient to inhibit the bacteria. For double-coated strawberries, CGPD1 and CGPD2 showed 1.6 and 2.5 log reductions, respectively, while control CGP showed a 0.6 log reduction. This indicates that the antimicrobial efficacy depends on the concentration of the extract added to the coating solution. Hashemi et al. [57] observed that the number of pathogenic bacteria including *S. aureus* in coated cucumbers decreased significantly as the concentration of saffron petal extracts integrated into the konjac glucomannan edible film were increased from 1 to 4%.

For triple-coated strawberries, the growth inhibition against *S. aureus* was significantly increased in all samples compared to the control, with CGPD1 having the greatest reduction (3.2 log CFU/g). Antimicrobial activity of CGPD2 was the most among double-coated strawberries, but not among triple-coated ones. This result might be because an excessive aggregation of antimicrobial substances caused by the formation of multiple layers which may prevent the release of the antimicrobial substance. These results indicate that antimicrobial effects of edible food coating can be determined by the type and amount of antimicrobial agents and polymers used, as well as other environmental factors.

## 4. Conclusions

The ternary blend film was developed using chitosan, gelatin, poly(vinyl alcohol), and a natural antimicrobial material such as *Duchesnea indica* extract using a casting method. *Duchesnea indica* extract showed an antimicrobial effect against both gram-positive (*B. cereus*, *S. aureus*, *L. monocytogenes*) and gram-negative (*S. enteritidis*, *E. coli*) bacteria. In agreement with the antimicrobial properties of the extract, composite film containing DIE showed a noticeable inhibitory effect on *S. aureus*, up to 4.9 log CFU/mL, by releasing DIE from the film matrix. The incorporation of DIE generally did not affect changes in mechanical and physical properties of composite films, while elongation at break decreased significantly with increasing DIE concentration. SEM results suggested a uniform distribution of DIE in CGP polymer matrices. The strawberry coating test showed that CGPD1 and CGPD2 inhibited up to 3.2 and 2.5 log CFU/g, respectively, against *S. aureus*. These results showed that the antimicrobial efficacy of strawberry coatings was affected by coating number, DIE concentration, and DIE interaction with CTS/GEL/PVA. The reduction in antimicrobial effect in the food coating test compared to that in composite films was considered a food matrix effect. Further research is needed on food packaging for extending the shelf life, as well as on antimicrobial mechanism and safety of DIE. Therefore, a ternary blend film incorporated with an antimicrobial natural extract, DIE, could be applied as a coating material in fresh produce, such as fruits, for controlling food-borne pathogenic bacteria contamination during the post-harvest processing.

## Figures and Tables

**Table 1:** Mechanical properties and water vapor permeability of films.

Film Sample	Thickness (mm)	TS (MPa)	EB (%)	WVP (g·mm·m^−2^·h^−1^·Pa^−1^)
CGP	0.153 ± 0.017 ^a^	17.72 ± 0.97 ^a^	106.43 ± 2.14 ^a^	5.16 ± 1.67 ^a^
CGPD1	0.178 ± 0.015 ^a^	18.65 ± 3.84 ^a^	87.44 ± 4.40 ^b^	6.47 ± 1.92 ^a^
CGPD2	0.162 ± 0.020 ^a^	21.59 ± 9.46 ^a^	62.24 ± 10.15 ^c^	5.30 ± 1.56 ^a^

^a,b,c^ Different small letters indicated significant differences among means in the same column (*p* < 0.05).

**Table 2:** MIC and MBC values of *Duchesnea indica* extract.

Bacteria	MIC (mg/mL)	MBC (mg/mL)
*B. cereus*	0.100	0.100
*S. enteritidis*	0.781	1.562
*E. coli*	1.562	3.125
*S. aureus*	1.562	1.562
*L. monocytogenes*	3.125	3.125

## Data Availability

The data presented in this study are available on request from the corresponding author.
